# On the Import of Symptoms

**Published:** 1868

**Authors:** Samuel Henry Dickson

**Affiliations:** Prof., etc., Jefferson Medical College, Philadelphia


### ARTICLE VII.

*On the Import of Symptoms.* By Samuel Henry Dickson, M. D., LL.D., Prof., etc., Jefferson Medical College, Philadelphia.

The relation of morbid phenomena to each other, and to the special forms of disease in which they manifest themselves, has always appeared to me to be a subject of deep interest, deserving the closest and most earnest inquiry. Pathologists are bound to seek with unwearied diligence and perseverance the cause and the history of every such change in the vital actions and conditions. The ultimate value of such knowledge cannot be over-estimated; but as yet, and in so far as our attainments have reached, we have been, in a degree at least, disappointed of the expected results.

In the Duke of Argyle's admirable work on "The Reign of Law," we are warned of the narrow limit set to our capacity, and reminded that the kinds of explanation which we are aiming to arrive at, are different in character and set apart. "That which is made plain to one faculty is not necessarily made plain to another. That which is a com-

*Since this memoir was drawn up, there has appeared in the Archives de Virchow, a memoir by Dr. Bernhardt upon the alterations of the stomach in poisoning by phosphorus.—This author quotes nearly all the observations published in France and Germany, in which the state of the digestive organs of those poisoned fatally by phosphorus had been noted. He adds to it some personal observations, and arrives at this conclusion: that phosphorus does not ordinarily determine inflammation of the gastric mucous membrane.

When this inflammation occurs, it must be attributed to the presence of gas in the stomach at the moment of the ingestion of the phosphorus. This body would then be oxydized, and would occasion the production of phosphorus and phosphoric acids, which alone could act as caustics.

plete answer to the question What, or to the question How, is no answer to the question Why. There are some philosophers who tell us that this last is a question which had better never be asked, because it is one to which nature gives no reply. If this be so," he remarks, "it is strange that nature should have given us the faculties which impel us to ask the question—ay, and to ask it more eagerly than any other. It is indeed true that there is a point beyond which we need not ask it, because the answer is inaccessible. But this is equally true of the questions What and How."

The inference to be drawn from these well-considered reflections is clearly that we must never cease to inquire, to ask, to explore. Only by the most persistent effort can we ascertain, if even thus, that we have reached the limits of the knowable in any direction. The philosophical physician, guided by these views, will refuse to regard diseases as mere "bundles of symptoms;" he will not be content to recognize merely their coincident presence; he will strive to detect the bond of connection which unites them, and to comprehend how and why they produce and depend on one another. He will be anxious to know both their relative and absolute value. He observes that a particular mode of suffering, headache, for example, is associated generally with fever; he finds it often connected with a local hyperæmia, congestive or inflammatory; but he soons discovers that this connection is not constant, and thus he is led to ask why it should occur in the individual case before him; or, reversing the problem, why it should not occur uniformly under contingencies apparently the same or similar.

Knowledge, such as is here implied, will not surely be long barren, but, when we acquire it, must abound with rich fruits. Symptoms will then be ever pregnant with meaning. They will teach us not only the existence and degree of intensity of morbid conditions, but will suggest to us their hidden nature and origin; and this apprehension of causes will not fail to lead us to the therapeutical arrest of consequences and effects.

At the present day, it must be confessed, we are sadly deficient in this department of our science. We deal habitually with symptoms as mere entities, sometimes associated, sometimes absolutely isolated. Of their roots we often know nothing, and are too apt to be content to know nothing. We appreciate them individually as we see them associated or concurrent with other symptoms, and then arrange them as parts of or belonging to certain diseases; but so loosely that we scarcely miss them when they do not attend, nor do we even allow their absence to suggest a question as to the character of the cases. We occasionally make an endeavor to separate and distinguish them as incidental or characteristic, uniform or uncertain, prognostic and diagnostic. But we decline to investigate, or we neglect to overlook their real significance, their relevancy, so to speak, to the underlying changes, the *causes* of which they are the out-cropping, superficial *effects*.

"Time is lost," says Theophilus Thompson, "in the laborious accumulation of miscellaneous facts. Numerism is productive in proportion to the intellectual intuition applied in the selection and appreciation of facts. There is an aristocracy of facts as well as of races, and the mind should be taught to discern their prerogative dignity. He who cannot or will not see that one fact is often worth a thousand as including them all within itself, and that it first makes all the other facts; who has not the head to comprehend, the soul to reverence a central observation or experiment, what the Greeks would have called a proto-phenomenon, will never receive an auspicious response from the oracle ot nature."

And Latham, following the same track of thought, argues, "There is nothing that we call symptom in disease which does not contain within itself much more than a mere sign; as dawn, a sign of the rising sun, is the effects of his beams; cloud above us, a sign of rain, is an actual gathering of the waters. Symptoms flow out of disease, being signs of something behind and beyond."

From our ignorance or carelessness in this matter arise obvious difficulties in our nosology and methods of classification; hence also modes of treating of disease absolutely paradoxical, as when we speak of fever without heat of skin, of small-pox without exanthem, and of cholera without discharges or spasm. We thus loosely acknowledge that we have not settled upon any indispensable symptom which shall be taken as the true, unfailing, and exclusive manifestation of the definite pathological condition. We regard an individual subject as an epileptic, because he *has had* convulsions; and we expect, though we cannot tell why diagnostically, that he will be again attacked. If he is not, we can no more explain his escape than his previous seizure.

In the Typhic group of fevers we recognize two, perhaps three varieties of " types," which we separate clearly enough. Each of these is marked by a characteristic eruption on which no little stress is laid. In true typhus, there are maculæ, dark and fixed, probably a deposit of pigment in the skin. In typhoid, a rose-colored erythema shows itself in defined circular spots, transient and in successive crops, a mere passing and local hyperæmia of small dermal vessels. In spotted fever, obscurely and capriciously combined (but what is *not* obscure in *its* history?) with a frequent, yet not uniform, cerebro-spinal meningitis, we have cuticular stains, blotches, petechiæ, and ecchymoses more or less diffused, varying in character, some of them slight erythema, some apparently pigmentary, and some truly hæmorrhagic.

Now, whatever else may be said of these cutaneous phenomena, whatever importance may be attached to them as prognostic or diagnostic, nothing even plausible enough to invite discussion has been offered in explanation of their presence—no reason given for their occurrence, no suggestion of the nature of their connection with the other symptoms with which they are concurrent or coincident, nor any conjecture of their meaning or purport.

Some have ventured to hint that they are exanthematous; but in the true exanthemata the cutaneous eruption is not only uniform and diagnostic, but it is somehow essential; it cannot be dispensed with; it must take place in a regular and consistent way; must go through its successive stages; if it be interfered with in its established progress, if it subside unduly, disappear, abort abruptly, "strike in," as the phrase is, the most serious consequences, as is well known, will follow, full of risk and evil.

But in the fevers of which we speak, the affection of the surface plays a very small and unimportant part. Spotted fever may exist with few or no spots; the patient dying with or without tetanus, with or without blood-stains, with or without meningitis. Typhus may always present its peculiar maculæ, but they are little considered either as diagnostic or prognostic; they can not be seen in the negro, and indeed we do not care much to look for them, as their quantity indicates nothing which it is of consequence to us to know.

A very different view, however, is taken by many modern writers, of the eruption in typhoid fever. Aitken says:

"The successive daily eruption of a few small, very slightly elevated,rose-colored spots,disappearing on pressure,each spot continuing visible for three or four days only, is peculiar to and absolutely diagnostic of typhoid fever." To make this dogma more emphatic, it is printed all in capitals. It is followed, however, by the statement that "the eruption is often so scanty that the physician may justly hesitate for a day or two to make a diagnosis;" and on the next page we find a conditional phrase which entirely destroys the force of his definition. "The eruption already described, and sudamina, are *nearly constant* in children after five years of age. "In children between one and five years of age the phenomena do not seem to be so easily observed as in adults." A symptom just stated to be "absolutely diagnostic," one "which clenches the diagnosis," is *nearly*, that is, not altogether, "*constant* in children after five years of age," and "not so easily observed in infants:" it is difficult to perceive why not; their skin is clear and delicate.

It is so hard to prove a negative that I will not contend for the possibility of a typhoid fever independent of the *tâche rouges*. But they are "often scanty," perhaps "not constant," undiscoverable in the negro, in whom we must diagnosticate the case without them. Sutton, in a good history of Epidemic of Typhoid in Kentucky, declares them to have been generally wanting; no one has drawn any inferences prognostically from their abscence or presence, abundance or sparseness. All that we know about them is that if from the sixth to the ninth day of a typhic fever, we find them on the body, we feel justified in giving name to an attack concerning which we may have previously doubted; we conclude further that diarrhœa impends, and meteorism, and bulging and ulceration of Peyer's patches, with risk of destruction of the patient by perforation through the coats of the intestine and the peritoneal serous tissue. But these are to us facts of simple coincidence; coming to us empirically by tradition and recorded experience. We have no guess at their connection with or relation to each other, nor at any condition serving as common cause to them all.

In the varied modes of death in these, and indeed in all fevers, widely considered, we are annoyed with further illustrations of the same profound ignorance. Our prognosis may be sagacious enough and generally correct; we may foretell the death, but too often we know not how or why it ensues. If it occurs early, we seldom find any thing to explain its necessity. We talk of congestion, and of toxæmia; but we know not why in certain cases the familiar congestion, so usually trancient, should persist fatally. Nor have we detected the blood-poisons, though sought for with the highest powers of the microscope and the most delicate chemical analysis. Of consequence we know literally nothing of the relation of the symptoms with the ultimate catastrophe.

When a death occurs from protracted fever, the usual lesions found are inflammatory or quasi-inflammatory. Does this prove the identity of fevers with the phlegmasia? Even if this were so, the nature and extent of such inflammatory lesions do not explain the death. Mere ulceration of the patches of Peyer heals in the majority of cases; it is only when it perforates into the peritoneum or a large vessel that we understand the result to have been of necessity fatal, as from hemorrhage in the latter case, in the former from nervous shock, perturbation, and prostration, for the patient dies far too promptly to allow any other explanation. In typhus the local lesions, it is conceded, do not account for the death.

We ask in vain why the black vomit of yellow fever is so terrible a portent of evil. If it be a hæmorrhage, as is so generally believed, surely the amount of blood lost in the majority of cases is not sufficient to destroy the subject. If it be a morbid secretion or excretion, its specific cause and mode of origin are most obscurely hidden, and the energy of that cause strangely capricious, for the result does not in any degree depend upon the quantity poured out, some patients recovering after profuse ejections of this sort, others dying with little or none. And what means the deep orange hue of the skin and eyes, met with so frequently as to give name to the disease, and yet not seldom wanting or scarcely observable either in the moribund or the convalescent? The characteristic fatty liver too, the *café au lait* or box wood atrophy, so rapidly developed, whence does it arise; what is its relevancy to the other conditions? Riddell tells us that a similar or analogous fatty degeneracy of the heart is even more uniformly present; if so, it is hard to say what is its origin or tendency, or how it appears in this combination.

But in truth, as we know little of the nature of life, we understand little or nothing of its extinction. Professor

Casper, of Berlin, a thorough expert in the autopsies of his Professorship of Medical Jurisprudence, tells us that "cases very frequently occur in which the most careful examination of the body can discern no material alteration that has any reference to the cause of the death of the individual. Cases of this kind can, as I have often seen, terribly perplex the inexperienced. Nothing abnormal in the surface of the body, nothing in the cranial cavity, nothing in the thorax, nothing in the abdomen. Of what did the deceased die?" (For. Med., vol. i, p. 56.) Elsewhere he says, (p. 59,) "In neuroparalytic death, not only is the mechanism of the body in no way altered, but there is also no perceptible change in its fluids or solids. It is of frequent occurrence," etc. We must accept unequivocally the conclusion he arrives at; that "the cause of death cannot with certainty be determined; this decision is in itself perfectly indisputable."

We boast, and with some justice, of the hygienic progress of civilization to which our profession has so largely contributed. Knowledge of causation is valuable as suggestive of prevention; but it is humiliating to reflect how little we know of, or knowing, how little we can do to counteract the causes of our endemics and our epidemics, more or less pestilential. Nay, with regard to attacks of pestilence continually occurring among us, sporadic or individual, how total our blindness! It is but the other day that two deaths occurred in this city, under circumstances deeply impressive, from what we call indifferently spotted fever or cerebrospinal meningitis. Two young men, members of the medical class of one of our colleges, having reached the termination ot the session in good health, one of them having passed honorably through his examinations, and waiting for his degree, both of them of good standing and well esteemed, of temperate and regular habits, residing not in the same house, but in the same street, three squares apart, following the same pursuits, but in no way associated together, were, within a few hours of each other, after no special exposure of any kind in either instance, under no discoverable excitement or annoyance—no ascertainable change or alteration of conditions—no undue or unaccustomed contingency—attacked suddenly, harshly, violently, and fatally. One of them lingered from Monday night, when he had a chill, until Thursday torenoon; the other sank within forty-eight hours from the moment of seizure. Both of them were fine specimens of youthful vigor; the second especially, was tall, manly, robust, strong, and active. The first was what is designated as a tetanoid case, yet not without abundant spots on the surface; the second was covered with purpuous blotches and ecchymoses, but did not suffer from spasm. There was no autopsy obtained. Now and then, for months past, the bills of mortality of the city would contain the notice of a death from this terrible disease, but for two weeks previous there had been no such record. The street is pleasant and well kept, the neighborhood healthy, both houses in good hygienic state, neat and clean, and the numerous residents in both of them free from every form of sickness or complaint.

In vain we endeavor to detect the source and nature of a poison so insidious, so rapid, so deadly; in vain we ask what determined its assault upon these two apparently firm and choice constitutions, or what lurking predispositions to special evil could be masked under such deceptive tokens of perfect health! Alas! of how little avail, how little instructive import, how little prophylactic importance, are the contingencies we are accustomed to dilate upon as of such high value in our disquisitions concerning hygiene, when we find that, under the most favorable circumstances of civilized and refined life, an obscure pestilence of the most incalculable and irresistible intensity can thus be generated among us, and select its victims by affinities which seem to contradict and run directly counter to all rational and preconceived anticipations.

Much stress has of late been laid upon the disappearance of the chlorides from the urine during the progress of pneumonia, and their return when convalescence is established. We are not clearly informed why nor where they are detained. They have not been detected in the inflamed parts, where Williams says they are arrested; nor is it known that they disappear from the renal secretion in any other of the phlegmasiæ. It is not shown that they come away with the sputa, where there is expectoration, nor what becomes of them in cases, occasionally met with, where there is none. Their restoration seems to attend or follow recovery of health, but whether to predict, nor denote, nor conduce to it in any sense.

The same may be said of the herpetic eruption which, occurring about the mouth, Todd regards as of favorable purport in pneumonia, and which some physicians and all nurses look upon as pleasantly prognostic in fevers generally; no one has even conjectured how or why.

A special form of disease is now recognized as "Addison's, or bronzed skin," of which the melainic or darkened color of the surface is considered to be the *sign*, whether *effect* or not, of a peculiar degeneration of the renal capsules. But many authorities, Virchow, himself a host, among them, deny the uniformity of the connection, and affirm that cases of varied degeneracy are met without the bronzing, and cases of bronzing where the capsules are found quite healthy.

Convulsion stands prominent as one of the most appalling of morbid phenomena. The hideous disfigurement of the "human face divine," the rapid contortion of the features and the grotesque grimaces, the rolling eye, the frequent strabismus, the gaping and shutting jaws, the protruded and wounded tongue, the bloody foam on the turgid lips, the twisted neck, the empurpled visage, the frame agitated with muscular contractions, clenching of the fists, bending and extension of the limbs, constitute a picture from which we shrink instinctively with pity and dismay. But what is the import, the meaning of this terrible display? I have gone through the painful details that I may impress on my reader's mind our total want of comprehension of the entire pathology of the case. It is not needed that I should remind him that these symptoms attend upon a prodigious variety of conditions of body and mind, and are not essentially dependent on, or connected with any one. "Epilepsy, as is well known," says Nannias, (Bouchardat, Annuaire, 1868, p. 196) "has sometimes its source in incurable material lesions. But it may happen that these lesions shall persist, and the fits disappear, so that we are forced to admit the intervention of another unknown element, upon which depends the appearance or disappearance of the epilepsy." Brown Sequard had indeed anticipated this view when he formed the abstract ontological idea of epilepsy, which, being present, his guinea pigs might be excited into a fit, but when it went away, were no longer excitable.

In our crude "bills of mortality" there is always found high up on the list, as far as numbers are concerned, the category of "convulsions." Now, nothing can be clearer than that this use of the term is a deplorable mistake—a grievous fault in our nomenclature, and one that should be corrected without delay. Convulsion is not, in any sense, a disease; it is a mere symptom, which occurs more or less frequently in the progress of every known form of disease. It is not uniformly associated with any, if we except puerperal eclampsia. It must not be confounded with spasm—tonic, or persistent contraction of contractile tissue—which constitutes, as we believe, the very essence of many diseases. Spasm is a frequent cause of death, but never appears by name on our bills of mortality. Convulsion cannot properly be said to cause death in any case, yet occupies a large space in them. Several forms of convulsion are physiological, and functionally available for useful purposes, as coughing, sneezing, vomiting. Spasm is invariably morbid and injurious. Convulsion is rarely, if ever, attended with pain, unless by undue protraction or peculiar violence; nay, it is sometimes pleasurable, as in laughing, and intensely so in venery. Spasm is always painful—the slightest cramp of a muscular fibre is hard to bear.

Convulsions often usher in attacks of disease, as of the exanthemata, pneumonia, etc., in children; the familiar chill of fever in adults as well is a mode of convulsion. They follow a fright, violent emotion, sudden shock; the irritation of worms in the intestines, of teeth in the gum, of sesamoid bones in the toe, may produce them, or a bit of glass imbedded in a nerve, a cut across the spinal cord, as in Brown Sequard's guinea pigs, or a tumor in the brain, the vertebral column, the liver. Sympathy and imitation alone will capriciously excite them; and, if we reason with Todd, they "eliminate by salutary explosion" certain poisons from the blood in some inconceivable way, as in cases of uræmia. I might go on to enumerate indefinitely additional occasions of their occurrence, as in the hysteric and pregnant woman, nervous, emotional, and mesmeric disturbances, but these shall suffice.

Let us demand, then, and most earnestly contend for the rejection of this heading from all municipal and general obituary records. I maintain that convulsion is not only not a disease, but it is not an essential portion of any disease, with the single and not perfectly clear exception above allowed, either characteristically or diagnostically. If the assertion of such essentiality be plausible in any instance, it would surely be so in epilepsy. But epilepsy is familiarly and by common consent divisible and divided under two heads, the characteristic condition underlying both of them equally and alike. One of them, known, I regret to say, by no English designation, but spoken of in French phrase as "le petit mal," exhibits no muscular agitation whatever. A lady riding does not fall from her saddle nor lose her bridle rein when attacked. Momentary vertigo, "vertige epileptique," a brief instant of mental inanity, vacuity, loss of consciousness, suspension of volition, of thought, this is all; there is nothing more recognizable either objectively or subjectively. Between the paroxysms, whether thus simply vertiginous or truly convulsive, we cannot consider the subject—how reasonably imagined in ancient times to be under demoniac possession!—we cannot regard him as free from disease. Yet what shall we say of its nature, its specific character, except that it is absolutely unknown? We have not detected the relation which it holds to any one among the numerous conditions with which we find convulsion to be familiarly associated.

There is another complicated question rapidly coming to press upon us as we acquire valuable accessions of knowledge from the kindred sciences. The microscope shows us every day new objects of investigation, entities, animal and vegetable, developed in diseased tissues, and in morbid secretions and excretions. Of some of these we know nothing except in this connection, we find them nowhere but in sick bodies. Others are found outside of the organism, into which they intrude themselves, and are detected by the mischief with which their presence is attended.

It is not always clear whether their development and growth are to be looked on as causative of the contingencies in which they make their appearance, or rather the effect or product of those contingencies, or perhaps, in fact, as mere coincidences, like certain other phenomena, to which reference is made above. Parisites attached to the living body, both entozoa and ectozoa, have been maintained to be altogether indifferent, innoxious, entirely consistent with good health; nay, Rush contended that worms in the human intestines played a useful part as scavengers. The various "grubs," as Wilson calls them, his entoza folliculorum and acari, and the several pediculi, whatever annoyance they may occasion, do not usually disturb the general system in any material degree. Nay, the trachina spiralis itself, recently noted so gloomily in our medical records, is now and then met with, as I have seen it, in the healthy muscles of a body accidentally killed,and displayed in our dissecton rooms.

But there are unnumbered tribes of vegetable and animalcular beings detected in the fluids and upon the tissues, which are alleged to be specific by histologists, and must at least be considered characteristic. Their absolute exclusiveness of appropriation is not yet made out satisfactorily. M. Simon is said to have "announced at a late meeting of the Pathological Society of London, for Dr. Hallier, of Jena, that he had, in the course of recent and very extensive investigations, discovered characteristic fungi in variola, variola ovinia, vaccinia, and in the blood in typhus, typhoid, and measles." Professor Salisbury ascribes intermittent fever to a palmella, and measles to an alga, and has detected characteristic fungi in syphilis and gonorrhœa. An ingenious paper from his hand in a late (April, 1868,) number of the American Journal of Medical Sciences, gives us the history and portraits of a penicillium, some toruli, several zymotoses, a botrytus, and a crypta, fungous parasites, and of animalculæ, a ciliaris, a trichina, and sarcina, and a trichomonas, all from the genito-urinary organs. The oidion and the leptothryx infesting the pseudo-plasm of diptheria, the algæ in muguet, in favus and porrigo—and I might adduce scores of other examples—have been subjects of dispute as to their relation to the morbidities always observed where they have been detected. Even in the halitus of pertussis, Poulet has found "a world of minute infusoria which were in all cases identical," the most numerous being bacteria, and a monas.

Still more obscure, if deeper obscurity be possible, are the relations of such morbid formation as tubercle and the cancer cell to the organisms which they seem to inquinate.—Vogel speaks of them as "individual," or rather "semi-individual," and there can be little doubt that they possess the property not only of taking root in a healthy part or tissue, and growing there, but besides and beyond this, they originate in that system, previously sound, a series of perverted actions which result in the formation and deposition of similar injurious pseudo-plasms, malignant cells and tumors in other parts.

Here then a wide field of controversy, doubt, and conjecture is opened to us. What is the true and available import of these discovered facts? Parasitic and quasi-parasitic diseases are communicable, and seem to be conveyed by means of the transfer of the palpable parasite, whether animal or vegetable, which migrates either spontaneously or with our help, and may be ingrafted. Such certainly is the fact with many or most of them, but we cannot venture, in the present state of knowledge, to affirm that it is true of all. With one remarkable exception, and some others imperfectly known and little considered, the diseases enumerated as of parasitic character and combination are among the contagious. But we are hardly prepared to say that the parasite is the materies morbi, the element of contagion, which is assumed indeed to be a poison or malignant force that in no distinct case we have ever detected as yet and separated. It will be very difficult to prove it to be the fons et origo mali; nay, in many instances it seems greatly more probable that we find it where it is, because the morbid surroundings afford it an appropriate nidus and pabulum.

The exception above alluded to is worthy of especial consideration. If the observations of Professor Salisbury upon intermittent fever be established as correct and well founded, we have a familiar malady parasitic but certainly not contagious; no one imagines the possibility of communication of malarial ague from a sick to a healthy man.

Yet it is not easy to conceive the existence of a parasitic alga or fungus, which shall bid defiance by its tenactity of life to so many perpetually recurring changes within the animal body for such indefinite periods of time, through so many alterations of season and climate, shall be capable of most abundant reproduction or multiplication within the organism, and such ready elimination with the several excreations, and still when passing out or ejected in notable amount, shall be totally incapable of finding its way into and impressing other animal bodies in its neighborhood.

And again, if these minute existences are not, all of them, like the palmella, intruders from without, how do they originate? Is Virchow right in his cell-pathology, maintaining that they are natural elements, cells of course, somewhat altered and modified merely by circumstances; or, as Paget and common sense teach us, new productions, altogether different from healthy material, and of essentially perverted and evil character? But if we press this inquiry we shall scarcely be able to refrain from intrenching upon the great biological controversy, now so hotly carried on by Pasteur and Donnè, and so many other renowned microscopists and histologists, concerning the mysteries of a disputed heterogenesis or "spontaneous" or "equivocal generation," in which, reluctant to engage, I decline to involve myself, and, therefore, close here somewhat abruptly this imperfect essay.

